# Are pads the priority? Contested framings of the Brazilian National Policy on menstrual health and dignity

**DOI:** 10.1016/j.wsif.2026.103298

**Published:** 2026-05

**Authors:** Isabela Hümmelgen, Inga T. Winkler

**Affiliations:** a SJD Candidate, Department of Legal Studies, Central European University, Quellenstr. 51, 1100, Vienna, Austria; b Associate Professor in Human Rights, Department of Social Sciences, Wageningen University, Hollandseweg 1, 6706 KN, Wageningen, Netherlands

**Keywords:** Menstrual health, Menstrual dignity, Menstrual movement, Public policy, Framing

## Abstract

Policymakers and policy advocates in Brazil have adopted various framings for menstrual policymaking, including menstrual precarity, health and dignity. Relying on the concept of framing and applying the “what is the problem represented to be?” approach, we unpack how menstrual needs are represented and how these representations inform and curtail proposed solutions in the Brazilian national policy. We rely on document analysis and interviews with activists and policymakers to present the debates from 2019 to 2024. We find that the Brazilian policy adopted novel and holistic language on health and dignity which is much broader than in other national contexts. Ultimately, however, policy priorities in Brazil mirrored those in other contexts and remain largely limited to distribution of menstrual products. Meanwhile, activists have been actively demanding the implementation of a broader agenda, that includes confronting menstrual stigma, investing in menstrual education, and providing health care services to menstruators, which opens up promising avenues for future policy directions.

## 1 Introduction

In October 2021, Jair Bolsonaro, then-President of Brazil, vetoed legislation on menstruation (Lei n. 14.214), which halted the provision of free menstrual products to women and girls in situations of vulnerability (Agência Senado, 2021). While the immediate impact meant that menstrual needs would remain unmet, the veto also catapulted menstruation to a central position in the country’s political and social debates. Menstrual activists catalyzed the demand around the country, advancing the agenda for provision of menstrual products (Caldeira, 2021; Dara, 2021) and pressuring Congress into reversing the veto, which eventually happened in March 2022. With Bolsonaro’s defeat in October 2022, the policy was to be implemented by the newly elected government of Luís Inacio “Lula” da Silva. In March 2023, Lula’s administration put forth its own version of the policy, the Decree n. 11.432/2023 (Decreto N° 11.432, de 8 de Março de 2023, 2023). Throughout this process, menstruation has been high on the political agenda in Brazil, leading to contestations about the scope and framing of policymaking.

Against this background, we pose two main questions: Which framings do various actors in the political landscape in Brazil adopt regarding menstrual needs? And how do these framings influence the national public policy? Relying on the concept of framing (Benford & Snow, 2000; van Hulst & Yanow, 2016) and applying the “what is the problem represented to be?” (WPR) approach (Bacchi, 2009), we unpack how menstrual needs are represented by the policy and discuss the potential pitfalls of the adopted strategies.

Public policies addressing menstrual needs in several other countries have been discussed in the literature. Scholars have shown how policies predominantly focus on menstrual hygiene and product distribution (Alhelou et al., 2021, 2022; King, 2024; McAllister et al., 2025; Olson et al., 2022; Thomson et al., 2019; Weiss-Wolf, 2020). For example, while the Scottish initiative of providing free menstrual products was applauded for its claim at universality, it does not address other menstrual needs (Bildhauer et al., 2022). In India, most policies have focused on material solutions, including improving water, sanitation and hygiene (WASH) infrastructure and access to products (Muralidharan et al., 2015; Patkar, 2020). Scholars have lamented that such material solutions do not address the underlying challenges rooted in menstrual stigma and gender-based discrimination (Alhelou et al., 2021), but may actually perpetuate these challenges by presenting menstruation as in need of concealment and control (Bobel, 2019; King, 2024; Lahiri-Dutt, 2015). Building on these findings, our premise is that meeting menstrual needs requires more than access to menstrual products, including comprehensive menstrual education, access to health care services, and challenging taboos and stigma.

Menstrual stigma is socio-culturally constructed and hinders discussion about menstruation, contributing to misconceptions and stereotypes (Johnston-Robledo & Chrisler, 2013; Olson et al., 2022). One example is the construction of menstruators as irrational or overly emotional, which can affect education, work and health (King, 2020). These impacts are perpetuated by limited menstrual literacy due to the lack of comprehensive menstrual education (Chandra-Mouli & Patel, 2020), even in medical and public health education (Martin, 2003; Sommer et al., 2020). While policies alone cannot undo menstrual stigma, they can play a role in reducing it by questioning social norms and reframing menstruation, for example, through education and awareness-raising (Olson et al., 2022). Therefore, the question arises whether policies address menstrual needs beyond the management of bleeding.

To date, research on menstrual policies has seen little attention in Latin America (Cifuentes Contador, 2025). Previous studies on the region have focused on the attitudes and experiences of specific populations with menstruation, such as young adults (Jones et al., 2025), children and adolescents (Borges et al., 2024; Pena et al., 2025), and low- and medium-income menstruators (Camas-Castillo et al., 2023). Studies in Brazil have engaged with the concept of “period poverty,” usually closely related to lack of access to hygiene products and potential health risks in vulnerable populations (Assad, 2021; Borges et al., 2024; Pena et al., 2025). In our study, we look at the process of policymaking in Brazil and critically question the notion of “period poverty” as limiting the proposed solutions set out in the policy.

The case of Brazil is significant not only because it brings a fresh regional perspective, but also because the policy’s framing explicitly refers to menstrual health and menstrual dignity. Precisely because developments in Brazil and Latin America have been somewhat insulated from the broader global discourse, it offers an opportunity to rethink menstruation as a policy field. Arguably, the region is less influenced by global health and development discourse and donor priorities than other regions (Patkar, 2020), which could allow for vernacular framings to develop. In terms of timing, menstruation began to gain traction a few years later than in other regions, in particular the early hubs in South Asia and Eastern Africa, which heavily relied on the framing of menstrual hygiene (Bobel, 2019; Cifuentes Contador, 2025). Linguistic differences with Spanish and Portuguese as dominant languages and English playing a lesser role further add to the potential for vernacular developments. It is in this context, that we examined the Brazilian policy which seeks to advance menstrual health and menstrual dignity—important framings for the Latin American context (Cifuentes Contador, 2025)—and thus promises a broader conceptualization of menstrual policymaking, only to ultimately deliver narrow solutions. Our study investigates the potential of the policy and seeks to explain why it has not (yet) fully delivered on its promise.

We examine how dominant and emerging framings by various actors, including policymakers, activists, the media and industry, influence the representation of the problem in national policymaking. We rely on document analysis and interviews to present the debates in Brazil and their development from the first proposals in 2019 to the policy implemented by Lula’s administration in 2023 and 2024. We identify the main actors and events that influenced the framings adopted to determine how the priorities came to be defined. We unpack these framings by considering the construction of meanings by different actors in the political landscape (Benford & Snow, 2000; van Hulst & Yanow, 2016). By applying the WPR approach, we critically consider the limitations and silences surrounding the policy and analyze if and how menstrual needs are met. We demonstrate that various actors relied on a range of different framings. More so than in other regional contexts, activists put forward a holistic understanding of menstrual needs, largely through the framing of menstrual dignity. Ultimately, however, compelling framings such as period poverty and menstrual hygiene resonated, precisely because they offer narrow solutions. The framings that prevailed in Brazil represented the problem as the lack of access to menstrual products, leading to the limited solution of product distribution to be prioritized by the national policy rather than tackling health-related issues, education, and menstrual stigma as originally promised by the framing of health and dignity.

Compared to other countries, the Brazilian policy adopted novel and holistic language. However, we find that despite this holistic framing, priorities in Brazil continued to be largely limited to product distribution. Although the legislation adopted the expression menstrual health, it conflated it with hygiene. Rather than addressing menstrual pain and health care needs, it reduced menstrual health needs to the management of bleeding, which reinforced stigmatizing narratives about menstruation. In addition, menstrual education, which is a primary focus of menstrual activists, was only recognized in vague terms with little guidance on promoting positive narratives about menstruation and confronting gender-based discrimination and stereotyping. Therefore, we argue that despite its novel framings the Brazilian national policy was narrow in its proposed solutions and lacked a comprehensive program that would ensure that no one is disadvantaged due to their menstrual status. This narrow approach to policymaking about menstrual needs has been observed in other contexts (Alhelou et al., 2021; Bildhauer et al., 2022; Scala, 2022), in which scholars have pointed out that distribution of products is not enough to guarantee menstruators’ health and well-being. In Brazil the framings of menstrual health and dignity suggested a stronger dialogue of policymakers with social justice and human rights movements that could have contributed to reaching different results. Our article traces why the limited solution of menstrual product distribution prevailed, even if activists advocated for broader priorities. We argue that the use of compelling, but limited framings in the media and beyond contributed to restricting the courses of actions included in the policy, undermining solutions that could tackle menstrual stigma.

In the following, we first provide an overview of the legislative proceedings that led to the policy’s elaboration. Then, we outline our methodology and analytical framework. To present and discuss our findings, we explore four dimensions of the policy and ongoing policy advocacy. First, we examine how the framing of *period poverty* came to be the dominant representation of the problem. Subsequently, we explore how the priorities set by the policy were constructed, explaining the meanings ascribed to *menstrual health*. Then, we explain how the framing of *menstrual dignity* as a positive lens to turn to solutions for unmet menstrual needs was interpreted. Finally, we discuss the need for compelling and persuasive framings to advance broader policy priorities.

## 2 Menstrual policies in Brazil

The first national legislative proposal addressing menstrual needs was initiated on 11 September 2019 by congresswoman Marília Arraes. She proposed legislation that would provide free access to menstrual products for school-aged girls (Projeto de Lei n. 4.968/2019, 2019). During the proceedings, at least ten other proposals were put forward by congresspeople of different political positions, leading to the full draft of the law proposal n. 4.968/2019, which focused on the provision of menstrual products to women in situations of socio-economic vulnerability. In August 2021, the proposal was approved by the lower house of Congress, subsequently endorsed by the Senate, and sent for presidential signature to be converted into law.

On 6 October 2021, Bolsonaro approved, signed, and published law n. 14.214/2021; however, he vetoed a substantial portion concerning the provision of menstrual products. He did not veto stipulations to “promote menstrual health” and “educate about menstruation” (Lei n. 14.214, de 6 de outubro de 2021, 2021). Although these indicate broad framing, they were not elaborated by the policy or supported by a comprehensive program and budget. The veto was met with strong rejection from civil society, who urged the government to offer an explanation—a task delegated to the then-Minister of the Woman, the Family, and Human Rights, Damares Alves. She reacted by asking the rhetorical question: “Is the priority vaccines or pads?” (Correio Braziliense, 2021), suggesting that during the COVID-19 pandemic there was no room to demand menstrual products. Meanwhile, the context of the health crisis contributed to the sense of emergency among menstrual movements, as activists were sensibilized to the lack of access to menstrual products by the most vulnerable population.

Urged by civil society’s reaction and pressure from social movements, on 14 October, congresswoman Erica Kokay filed a petition for Congress to re-discuss, review, and potentially defeat the presidential veto (Kokay, 2021). On 10 March 2022, Congress defeated the veto, including with the support of pro-government representatives, and law n. 14.214/2022 was republished in its full original contents. Nevertheless, this law was never implemented by Bolsonaro’s government, which made no efforts to develop the necessary programs.

Implementation of the program only began after President Lula took office in 2023. In March, he signed decree n. 11.432/2023 (Decreto N° 11.432, de 8 de Março de 2023, 2023), which established the Program for Protection and Promotion of Menstrual Health and Menstrual Dignity and set the objective to “ensure the free provision of sanitary pads and other basic menstrual health care, with the aim to promote menstrual dignity” (Decreto N° 11.432, de 8 de Março de 2023, 2023). According to the policy, menstrual products would be provided through the public health system, public schools, and the prison system. In February 2024, the government announced that free distribution would also take place through the *Farmácia Popular* [Popular Pharmacy], which are subsidized drugstores to provide free or discounted medication (Ministério da Saúde, 2024a, 2024b; Secretaria de Comunicação Social, 2024). According to data provided by the government, pads are available in more 31,000 Popular Pharmacies targeting 24 million people (Ministério da Saúde, 2024c).

## 3 Research design and methodology

To investigate the debates surrounding policymaking in Brazil, we used document analysis and interviews. We analyzed legal documents and public statements of political actors in the legislative and executive arenas, including law proposals, reports on legislative proceedings, official statements, and media interviews. All documents and statements are publicly accessible and most of them were taken from government websites, apart from the statements, for which we used media sources. Documents and statements were selected based on their relevancy to understanding the current legislation approved and implemented and its proceedings in parliament and in the executive. We also included reports and studies produced by civil society, if relevant to the context, resulting in a total of around 70 documents. Documents are cited throughout the article to explain the policymaking process, the framings used, and the perspectives of different actors involved in the process.

In addition, we conducted 24 semi-structured interviews with menstrual activists, policymakers and members of their staff, and other relevant actors. Participants resided in 13 different Brazilian states, plus the Federal District. One participant identified as cis man, 23 identified as cis women. Besides policymakers and members of their staff, other participants were part of the menstrual movement. They either actively participated as members, coordinators, or founders of local organizations, or considered themselves independent activists and researchers who collaborated with different organizations. One participant worked for a brand of menstrual products and was interviewed for their role in promoting policies in Brazil. An initial sample of participants was selected based on organizations cited by policymakers in legal proceedings, complemented by online searches for organizations that work in field, and the remaining participants were reached through snowballing. They were included in the study if we evaluated their work and experiences to be relevant to understand the context of policymaking in Brazil. Participants were contacted based on contact information provided in public websites or by other participants. The interviews were conducted between July 2023 and April 2024 using the video-conferencing software Zoom. All communication with participants was in Portuguese. Interviews lasted around one hour. Questions focused on the participants’ activities in the menstrual space, their opinions and experiences with policymaking, and their insights into menstrual activism and other actors in the field, such as industry and the media. All the participants were informed of the conditions of participation in the study and provided their informed consent. We received ethical clearance to conduct the study from the Ethical Research Committee of the Central European University.

We carried out a qualitative thematic analysis of all documents and interviews (Braun & Clarke, 2022), using the software NVivo. We developed a codebook following a deductive-inductive approach, based on the common themes that we identified in the interviews and the existing literature on menstrual policies. The codes covered the expressions used by interviewees, such as period poverty, menstrual dignity and menstrual health, and their meanings and understandings. They also captured the participants’ opinions of the public policies and their implementation under Bolsonaro’s and Lula’s administrations. We coded the interviews in Portuguese and translated direct quotes used in this article into English.

Our study faced several limitations. First, it focused on national policy, only addressing state and municipal policies where they relate to the national debate. It was not our aim to provide a comprehensive overview of policies related to menstruation in Brazil, but to investigate how different actors and their framings shape the national narrative and priorities around menstruation. Second, some key policymakers were not available for interviews. We complemented the interview data by accessing public documents, including their speeches given in Congress. Therefore, despite the limitations, the combination of documents and interviews generated rich insights into how the Brazilian menstrual policy debate unfolded.

## 4 Analytical framework

To unpack how menstruation and menstrual needs are represented, we draw on the concept of framing. Given our interest in the interaction between policy advocacy and policymaking, we rely on social movement scholarship as well as public policy literature. With regard to social movements, framing serves to explain their courses of action and motivations (Benford & Snow, 2000; Snow et al., 1986). Robert Benford and David Snow (2000, pp. 613–614) define framing as “signifying work or meaning construction” that social movements engage with in the process of collective action. They (2000, p. 614) suggest that framings mobilized by social movements are “action-oriented sets of beliefs and meanings that inspire and legitimate the activities and campaigns.”

Public policy scholars have built on the work of Schön and Rein (1994) to develop a process-oriented and politically-relevant notion of framing (van Hulst & Yanow, 2016). They stress that *framing* is more than a *frame*, it is a process of meaning-making and meaning dissemination that involves sense-making, selecting, naming and categorizing, and storytelling (van Hulst & Yanow, 2016, p. 96). In what follows, we explore how menstrual needs are framed by political actors and activists in Brazil and how they mobilize different ways of identifying the problems and proposing solutions.

To conduct the analysis of the policy, its effects and limitations, we applied the “what is the problem represented to be?” (WPR) approach, developed by Carol Bacchi (2009). The WPR approach serves as a tool to understand how policy is shaped by public discourse, including narratives circulated by civil society, social movements, the media and industry (Crammond & Carey, 2017, p. 407). While the conventional approach to analyzing policymaking examines how governments react to exogenous problems, Bacchi argues that governments actively produce the representation of the problems to be tackled by policies (Bacchi, 2009, p. 1). This approach therefore serves to question taken-for-granted assumptions to identify conceptual premises within problem representations (Bacchi, 2009, pp. xv, xix; Bletsas, 2012, p. 43).

Based on this framework, we sought to comprehend what was identified as the problem by policymakers and activists and how its representation was built in the political and social spheres. We started from the concrete interventions proposed by the law and used the public documents and interviews to determine how and why they were considered the most appropriate solution to address menstrual needs in Brazil. We applied the approach suggested by Bacchi to unravel the underlying assumptions. We also considered the silences and limitations of the policy and developed suggestions for more comprehensive future policies.

## 5 Findings and discussion: menstrual precarity, health and dignity

In this section of the paper, we analyze how different framings of menstrual needs have shaped the debate and policy priorities in Brazil, discussing the framings of menstrual precarity, menstrual health, and menstrual dignity.

### 5.1 Identifying the problem: menstrual precarity

Public discourse in Brazil focuses on the framing of period poverty-—usually referred to as menstrual poverty or menstrual precarity. These expressions are frequently reproduced by the media, the industry, policymakers, and to some extent activists. From its first conception, the Brazilian legislation foregrounds menstrual precarity, which it portrays as affecting women and girls in situations of socio-economic vulnerability. The policy’s goal is to eradicate menstrual precarity. First introduced in PL n. 4968/2019 (Projeto de Lei n. 4.968/2019, 2019, art. 2, I), and later incorporated into the law, menstrual precarity is defined as the “lack of access to hygiene products and other necessary items during the period of menstruation, or lack of resources that facilitate acquiring them” (Lei n. 14.214, de 6 de outubro de 2021, 2021). The problem tackled by the policy is represented to be the lack of access to products.

As the main policy frame, this definition is reductionist. Many activists argued that period poverty is related to a lack of access to resources, including menstrual products and hygiene items such as soap and toilet paper, but also to shortcomings in menstrual education, information, WASH infrastructure, and safety (INT12, INT13, INT16, INT21, INT22, INT24). Scholars, too, have argued that period poverty involves complex, multidimensional circumstances encompassing scarce water and sanitation infrastructure, insufficient waste disposal, social stigmas, and inadequate education (Assad, 2021; Bant et al., 2021).

Nevertheless, the immediate connotation of the term poverty is material deprivation, and this has been the main emphasis of congresspeople and the central framing of the law. Other factors, such as access to water and sanitation, were briefly mentioned during legal proceedings but not included in the final text. Mostly, debates in Congress described how menstruators need to resort to unsafe methods to manage their periods (using paper, plastic bags, socks, and bread crumbs to contain the blood) (Assad, 2021, pp. 142; 146), which may result in health problems.

Period poverty is invoked with a sensationalist appeal that sustains the need for a policy. Indeed, Arraes justified the use of the term by claiming that period poverty is a real problem for teenagers representing risks to their health and leading to school absenteeism (Projeto de Lei n. 4.968/2019, 2019, p. 2). Building on this justification, in their proposals (n. 1547/2021; n. 1807/2021) Severino Pessoa, Bira do Pindaré and Lídice da Mata explained that not having the means to properly manage the bleeding can increase the risk of infections, skin irritation, vaginal itching, discharge, as well as mental health conditions, including anxiety and depression (Arraes et al., 2021, pp. 23; 32). They argued that, by needing to adopt unsafe methods, menstruators cannot fully enjoy their rights to health and dignity. As pointed out by Chris Bobel, such approaches are common in policymaking and advocacy to bring attention to unsavory—and in reality unlikely—materials to collect blood to justify programs for product distribution (Bobel, 2019, pp. 116, 121). This highlights the importance of stories as part of framing as they serve to present meaning in coherent and graspable ways tying the perceived problem together in a persuasive way (van Hulst & Yanow, 2016: 100–101).

The circulated stories about girls having to turn to cow dung, breadcrumbs or rags—not cloth—present a persuasive framing that resonates with the public, the media and with policymakers. It is combined with frequent references to girls missing schools due to lack of access to menstrual products—a claim that has been debunked repeatedly as the realities of school absenteeism and associated reasons are far more complex (Austrian et al., 2021; Nelson et al., 2025). This framing has crossed into sensationalization, and due to its reductionism it leads to “quick, technical fixes” reduced to menstrual products, while ignoring the complexity of the challenges related to menstruation (Alhelou et al., 2021, p. 10). This perspective reinforces the notion of menstruation as a shameful occurrence to be concealed and controlled (Olson et al., 2022, p. 3; Røstvik, 2022, p. 62). Menstruators continue to receive messaging that menstruation requires self-surveillance and self-disciplining, which is detrimental for their participation in society (Wood, 2020).

Activists further criticized the framing of period poverty because it risks stigmatizing people living in poverty. Úrsula Maschette Santos, a psychologist and specialist in menstrual and cyclical health, shared that “this term has also been problematized quite a lot. Because it reduces poverty to the [individual] body.” Similarly, Juliana de Souza Gonçalves, co-founder of the Rebbú Institute, even though she uses the term in her activism, acknowledged that period poverty creates an us-and-them divide conveying that “you have a certain superiority, that they are not worthy.” Gonçalves referred to sustained beliefs that see living in poverty as being ‘unworthy’ and ‘inferior’ in terms of personal achievements.

Overall, although the term period poverty was criticized, it remained widely present in the vocabulary used by activists and policymakers. When prompted to explain its use, interviewees said that it is the most well-known term, especially among government officials (INT08, INT09, INT12, INT13). It is “easily understood” and can be impactful, because it is related to poverty itself, which is a common focus of policymaking (INT08, INT09). Jumara Souza Pimenta, coordinator of the project *MenstRUA* [Menstruate], explained that she does not like the term, but “we know that somehow these are terms that still attract a lot of attention from public policy, right?” Given that almost anyone who menstruates is familiar with the experience and distress of not having menstrual products at hand, a frame that presents this experience as a recurring one has high experiential commensurability, as termed by Snow and Benford (1988, p. 208). Recognizing the importance of frame resonance, activists have chosen a compelling and persuasive—albeit limiting—framing.

Applying the WPR approach, we traced how the representation of the problem as period poverty rose to prominence. We identified the role of industry actors and the media in reproducing it. In 2021, a study on the topic was funded by Procter & Gamble (P&G) and its brand “Always,” which was running a campaign donating pads. To better understand whom to target with donations, P&G funded a study to determine menstrual needs in Brazil (INT 04). This study concluded that “1 in every 4 women has missed out on school because she could not buy pads” (Always, 2024) and reported that 50% of respondents at some point had to replace a pad with toilet paper, old clothes or paper towels. It also labelled the problem as period poverty. Maschette Santos elaborated how the industry played a role in dictating the representation of the problem—a problem to which they could provide solutions with their own products:

One in four girls can’t afford to buy sanitary pads. One in four girls miss school because they can’t buy sanitary pads. So if you can’t buy them, then donating them can solve the problem. … So they [the brand Always] can respond to the two problems they encountered here in this data with their own product. … So there is a very important warning, which is that there is no data from the public sector, the government does not have reliable data. It allowed the private sector to dictate … where things should go. (Maschette Santos, interview with the authors).

Indeed, the menstrual care industry has a powerful role in determining how menstruators choose to manage their cycles (Punzi & Werner, 2020, p. 834). They promote a consumerist relationship to menstruation, in which commercial menstrual products—and particularly pads—are marketed as not only effective and comfortable, but also as the only clean and hygienic way to manage menstruation, thus reinforcing the imperative to conceal menstruation (Kissling, 2006, p. 123; Lahiri-Dutt, 2015, p. 1169; Vostral, 2008). Ultimately, the solution provided by multinational corporations relies on the commodification of the menstrual cycle, eclipsing alternative ways of handling bodily fluids (Lahiri-Dutt, 2015, p. 1169; Punzi & Werner, 2020). In their analysis of the Scottish policy, Bettina Bildhauer and others come to a similar conclusion: by framing the problem to be solved as period poverty, the law places emphasis on commercial products (2022).

As mentioned by Maschette Santos, P&G played a key role in Brazil in framing the perceived problem as related to unmet menstrual needs (lack of products) and providing the solution for this problem (their own products). By commissioning and publicizing the study, they presented a narrative aligned with their own marketing interests. Daniela Gil Rios, Director of Government Relations and Public Policy at P&G, acting in her personal capacity, recalled how she disseminated the results of the study to government, policymakers, and NGOs, because she wanted to prompt action: “I must have sent more than eighty emails and messages, based on this mapping of our studies, showing this reality that the girls hide what they use to replace [menstrual products, such as] bread crumbs, newspaper and fabric scraps.”

The results from P&G’s study became the main data reproduced to address period poverty. Based on this study, period poverty made the headlines of a major TV reporting program, Fantástico. In May 2021, Fantástico presented period poverty as an urgent matter, reinforced its definition as the lack of access to menstrual products, and cited P&G’s study to support its narrative (Globoplay, 2021). This report brought the topic to national attention for the first time (INT 10, INT23). According to Flávia Regina Marques Castelhano, who is a researcher and cofounder of the *Coalizão por Dignidade Menstrual* [Coalition for Menstrual Dignity], P&G’s study “was brilliant because they managed to gain a lot of media attention. Then you see all the headlines. One in four girls, one in four girls and so on. This ended up focusing a lot on the issue of the lack of sanitary pads.” With that, the brand’s study became a central reference point cited by policymakers, activists, and the media (INT08, INT22). The story that gained traction was the sensationalized account of girls’ unmet menstrual needs. The study cemented the framing of menstrual poverty as the problem to be addressed. The narrow selection of the issue as part of the framing process was made compelling through media accounts and storytelling.

### 5.2 Setting policy priorities: conflating health with hygiene

In response to the identified problem of menstrual precarity, the policy approved by Congress in 2021 and developed by Lula’s government in 2023 sought to implement a menstrual health program. How the term “menstrual health” is used in policy documents further illustrates that framing means selecting; and here, the selection process resulted in a narrow understanding of menstrual health. While the policy’s title raises the expectation of comprehensively addressing the health of menstruators, the policy’s actual—and only—goal was the distribution of menstrual products. The congressional proceedings, accompanied by the media, focused on hygiene standards and health issues resulting from lack of products. In the lead-up to law n. 14.214/2021, all proposals brought forth by congresspeople related to the provision of menstrual pads (Arraes et al., 2021). They argued that the lack of menstrual products was detrimental to menstruators’ physical health (being a cause of diseases and infections) and mental health (being a cause of anxiety and distress) (Arraes et al., 2021). With that, the idea of menstrual health was conflated with the need to promote hygiene by providing products. The underlying problem, namely that menstruation and the fear of ‘leaking’ or ‘staining’ cause anxiety in menstruators, remained unaddressed. Monica Crisostomo Johnston, who was an aide in Arraes’s office, explained that they used menstrual health during the legislative proceedings because “this is a matter of public health.” Arraes used the same expression in one of her speeches in Congress. Crisostomo Johnston explained that they researched data in the public health system about women who had health problems related to lack of menstrual products:

We collected data, for example, of women who have problems … In the year 2015 we had a percentage of thirty-four, thirty-five percent of the total number of women treated by the SUS [public health system] with uterine problems, was due to the lack of menstrual items, menstrual dignity. Women did not have access to tampons or pads, so they used newspaper, whatever you can imagine, corn, bread … This caused health problems. (Crisostomo Johnston, interview with the authors)

Some research has explored the link between inadequate menstrual hygiene and infections (Borg et al., 2023; Sumpter & Torondel, 2013), but there is no conclusive evidence that the lack of access to menstrual products causes health issues (Bobel, 2019, p. 122). In a systematic review on intimate hygiene practices and reproductive tract infections, Daher et al. (2022) found some evidence of unhygienic practices increasing the risk of infections, but they stressed that excessive hygiene practices that may alter the vaginal pH or vaginal microbiota also lead to a higher risk of infections. Considering the lack of conclusive evidence, the narrative that relates menstrual health to inadequate use of materials to collect blood seems to have been overstated in Brazil, contributing to the risk of sensationalization based on reductionist assumptions.

Among menstrual scholars and activists, menstrual health has a broader meaning beyond the management of bleeding and ensuring hygiene to reduce the risk of infections. In the definition elaborated by Hennegan et al., menstrual health is defined as “a state of complete physical, mental, and social well-being and not merely the absence of disease or infirmity, in relation to the menstrual cycle” (Hennegan et al., 2021, p. 2). Brazilian activists highlighted how, in their view, menstrual health also includes menstrual education and access to health services (INT06, INT14). They related the term to self-awareness about the body, knowing what is healthy and identifying any symptoms or signs of distress and discomfort (INT18). Larissa Agostini de Souza, an independent activist and member of the *Partido Socialista Brasileiro* [Brazilian Socialist Party], explained why and in which contexts she sees this term as useful:

There’s a very positive aspect to bringing up this health issue. To reinforce that yes, menstruation is health, not a disease, right? It’s a vital part of the body’s functioning. Also, so we can advance in other issues about endometriosis, PMDD … which is super neglected, hidden, most doctors don’t even recognize it; they don’t know because we have this whole stigma around PMS. (Agostini de Souza, interview with the authors)

In the process of designing the policy, most of these health dimensions were left silenced. Policymakers seemed to assume that access to menstrual products was the only measure necessary for public health. There was no mention of health conditions associated with menstruation, such as Heavy Menstrual Bleeding, Polycystic Ovary Syndrome or endometriosis, or suggested measures to improve diagnoses and health care for menstruators who face pain and discomfort. None of the proposals encompassed a comprehensive policy for menstrual education and awareness-raising, even though some congresspeople mentioned that taboos and stigmas should be addressed. This demonstrates that once a compelling frame has been created, there is a tendency to stick with the narrow framing.

In the following years, some initiatives have sought to expand the menstrual health agenda. Many law proposals related to the topic of menstruation are waiting to be discussed in Congress. One relevant discussion centers on menstrual leave, with proposal n. 1.249/2022 by Jandira Feghali taking the lead. Feghali suggests that legislation should guarantee “three consecutive days of leave, each month, to women who demonstrate severe symptoms associated with menstrual flow” (Projeto de Lei n. 1.249/2022, 2022). Endometriosis has also received some attention by congresspeople, with a leading proposal (n. 1.069/2023) by Silvye Alves suggesting legislation that establishes “basic guidelines for improving the health of women with endometriosis, [and] includes endometriosis with disabling manifestations in the list of diseases that do not require a qualifying period for the granting of sickness benefit and disability retirement” (Projeto de Lei n. 1.069/2023, 2024). However, thus far, none of these proposals have advanced to be voted on by Congress or are being discussed by the public.

Advancing menstrual health requires the elaboration of a holistic program with menstruators’ needs at its core, which goes far beyond the limited notion of menstrual health as conceptualized in the Brazilian policy. While the content of such a holistic policy has to some extent been determined, essential steps in the framing process are thus far missing for meaning-making. Selecting the issues, categorizing them and packaging them through storytelling would be essential for this broader framing to resonate.

### 5.3 Proposing solutions: menstrual dignity

Apart from menstrual health, the most widely adopted framing to overcome the problem of period poverty in Brazil was menstrual dignity. The 2023 policy explicitly referred to promoting menstrual dignity, and activists commonly relied on the framing, which has been gaining traction in Latin America. As explained by Marques Castelhano, “our theme is menstrual dignity, that comes from a problem, the problem is period poverty.” Activists understood that if a person cannot menstruate “in a dignified way” they live in a situation of menstrual precarity (INT03, INT11, INT23). To menstruate “in a dignified way” is to not feel any discomfort during one’s period (INT03) and to have access to all the resources necessary, including menstrual products and WASH infrastructure (INT07, INT14, INT19). In this regard, menstrual dignity still seems to be related to menstrual management. This framing suggests that menstruators’ dignity is hindered by lack of proper infrastructure and resources, which means dignity is to be attained through better conditions for period management (Zivi, 2020, p. 127). Only if menstruation is controlled can dignity be restored, so the reasoning. This idea of menstrual dignity as something to be attained through ‘proper’ menstrual management, privacy and control has been challenged by critical scholars (Bobel, 2019, p. 211).

Yet, menstrual dignity goes beyond the material dimensions of menstruation (INT08). Together with other organizations, Instituto Rebbú developed a manifesto, which they proclaimed “is not about pads, it is about menstrual dignity.” The document explained that menstrual dignity is also “about the right to information and choice. It is also about being able to talk openly about the topic, without judgement or prejudice. It is knowing your own body … It is about ensuring that having a uterus does not become a barrier to personal and professional development” (Instituto Rebbú, 2023). Fabíola Magnago Mozine, coordinator of the project *Segue o Fluxo* [Follow the Flow], elaborated that “we use the term menstrual dignity for a political reason, a question of not simplifying the issue to just pads.” It is connected to an idea of rights (INT10), bodily autonomy and access to information (INT06, INT07). The dignity frame, besides being a means to capture attention (Bobel, 2019, p. 216), also accesses the discursive level of how menstruation is represented in the public sphere. By relating menstrual needs to ideas of rights and dignity activists call for a holistic recognition of menstrual needs and refuse to reduce menstruators to unorderly bodies that have no place in public space and discussion (Zivi, 2020, pp. 136–137). Mariane Marçal do Nascimento, who is a project coordination assistant at Criola, defined menstrual dignity within the frame of justice: “menstrual dignity has a lot to do with this counterpoint to what we have as undignified, indignity. … It could also be used as we do in reproductive justice, it is menstrual injustice.” Indeed, dignity invokes the connection to rights and justice. Although activists considered all these dimensions and argued that menstruation “impacts every area of our lives” (Instituto Rebbú, 2023), they hardly formulated concrete policy demands. The “more than pads” was rarely defined and translated into concrete steps.

At the policy level, the decree signed by Lula included menstrual dignity as the title of the program and the government’s website elaborated that the program “promotes health of those who menstruate and provides them with the opportunity to access spaces and other rights without restrictions.” It also promoted “gender equality, social justice, education and human rights.” (Ministério da Saúde, 2024d). However, these broader concerns are not accompanied by concrete measures, which results in a dissonance between the meanings attributed to menstrual dignity and the actions taken to ensure its realization. While the issue of menstrual dignity has been named in the framing process, the “more than pads” has not gone through the process of meaning-making, selecting, categorizing and storytelling. Whereas stories of menstruators having to rely on “undignified” ways of managing their bleeding are extremely compelling, other issues have found little resonance because they remain too vague and abstract.

Overall, the framing of menstrual dignity seeks to expand the representation of the problem beyond the lack of products. Driven by activists, this framing includes the dimensions of human rights and justice in the debate. It reflects the willingness of the movement to direct its efforts beyond material solutions. However, to date, little of this recognition of the problem has been translated into concrete policy actions. The lack of resonance may be attributed to the incomplete framing process which has not gone beyond naming the problem.

### 5.4 Advocating for alternatives: beyond product distribution

When considering the notions of menstrual health and menstrual dignity, Elaine Amazonas, project manager at Plan International Brazil in Bahia, for instance, reflected that “distributing sanitary pads, it’s important, but it doesn’t encompass menstrual health or dignity.” Marçal do Nascimento shared how she believed the policy can be furthered within the framings of menstrual health and dignity:

The program, as the name suggests, is the program for the protection and promotion of menstrual health and dignity. It is not a program for distributing sanitary pads, right? There are other premises that are part of this policy. … it is about providing education, it is about providing information. … There is the taboo issue … of talking about menstrual blood. (Marçal do Nascimento, interview with the authors)

In terms of menstrual health, activists demanded policies that include access to health care. Policies should be concerned with specific health conditions to facilitate accurate diagnosis, expansion of the network of doctors and health professionals who are sensitive to issues related to menstruation, and provision of treatment and resources for menstrual pain and symptoms (INT03, INT06, INT08, INT18).

The activists’ main demand was the provision of menstrual education and awareness raising about menstruation. Menstrual education should be included transversally in school curricula, in addition to more comprehensive sex education (INT06, INT08, INT11, INT14). According to de Souza Gonçalves, the issues need to be interconnected: “within this education for gender equality there would be the issue of menstrual dignity … I also think sex education … and gender identity, because … one thing is linked to the other.” Besides the emphasis on schools, interviewees highlighted the need for menstrual education in other contexts, such as universities, the health care system and the carceral system, including for non-menstruators (INT11, INT12, INT14, INT17). In the words of Souza Pimenta, “we’re not going to limit this topic to only people who are menstruating.” What we see here is the beginning of the framing process to redefine menstrual health and dignity as “more than pads” by formulating and conveying concrete demands and suggestions.

Given the disconnect between the policy’s narrow implementation of solutions to meet menstrual needs and its broader title and the existing suggestions of broader policy alternatives, the WPR approach calls for evaluating why these alternatives have not found resonance. Bacchi stresses that there are commonly competing representations of the problem, which means that the policy could have developed differently (Bacchi, 2012, p. 10). Agostini de Souza shared her impression that more complete proposals are more difficult to get approved. She recalled the program *Menstruação sem tabu* [Menstruation without taboo], a comprehensive program proposed in the state of São Paulo in 2019. Apart from the distribution of products, it included measures to confront discrimination related to menstruation, distribution of materials about the topic, and incentives to hold events that normalize menstruation (Projeto de Lei n^◦^ 1.177, de 2019, 2019). After being introduced by a broad coalition of political parties, the proposal was approved by the state assembly, only to be vetoed by governor Tarcísio de Freitas in 2023, who argued that there is a “solid state public policy regarding overcoming menstrual poverty, which includes access to necessary supplies, such as sanitary pads, as well as information about the menstrual cycle” (Bronze and Giovanna., 2023), demonstrating once again the reductionism in understanding menstrual needs merely as the management of bleeding. Agostini de Souza pondered that “it’s one of those cases where it wasn’t approved [because] you have a person who doesn’t understand anything. He thinks that when he hears the word menstruation [in one policy], that is it, the issue is resolved.” She criticized the governor’s lack of understanding that more than the distribution of products is needed for comprehensive policies. The São Paulo proposal is an example of policymaking that intended to be comprehensive but fell short in communicating its intention during political negotiations. Governor de Freitas used the reductionist narrative about menstrual needs to veto a program that had the potential of being the most comprehensive in the country. The original framing was so persuasive that it stuck and limited the space for more expansive framings to take hold.

Still, expanding the understanding of menstrual health and dignity can lead to more holistic policy priorities. Overall, menstrual activists demand policies that represent a shift from hygiene-oriented actions to a more holistic understanding of menstrual health and dignity, which includes education, research, training, and access to health care. The articulation of these comprehensive demands by menstrual activists may be the most distinctive and promising feature of Brazil as a case study when compared to other regions, in which not just policy-makers but also advocates formulated their demands in a much more limited way (Alhelou et al., 2021; Bobel, 2019; Weiss-Wolf, 2020). The Brazilian policy already points in the direction of more holistic policy development by mentioning menstrual health and education. However, implementing these measures will require compelling narratives illustrated through stories that demonstrate the need for comprehensive menstrual policies.

## 6 Conclusion

In this article, we explored the framings surrounding the national policy on menstrual health and dignity in Brazil and critically discussed how they have contributed to proposed solutions. We found that the policy has sparked conversation about menstruation in the country and has pushed the agenda forward leading to positive change in menstruators’ lives. However, we found that it did not implement any proposals for confronting taboos and stigma, envisioning proper education for the population, or promoting care for menstrual health conditions. Applying the WPR approach, we explained how the limited solutions resulted from the narrow representation of the problem as a lack of menstrual products, which is assumed to be the cause of school absenteeism, inadequate hygiene standards, and health issues for menstruators.

Based on our analysis of documents and interviews, we concluded that although the discussions are permeated by broader terminology (such as health and dignity), compelling framings such as menstrual precarity and hygiene limit the policy’s impact by focusing almost exclusively on the solution of product distribution. We found that the engagement of actors such as the media and the industry contributed to the prevalence of the menstrual precarity framing and the representation of the problem as the lack of access to products. While the framing of the lack of menstrual products presenting a problem for the realization of menstrual health and dignity proved to be salient and powerful, the broader framing of “more than pads” was not. This may be attributed to missing essential steps in the framing process to move from abstract concepts to concrete and compelling frames.

This narrow focus is not unique to Brazil. Policies focused on the material aspects of menstruation, such as distribution of products, reduction or elimination of taxation, and investment in the WASH sector have been the mainstay of policies addressing menstrual needs worldwide (Alhelou et al., 2021; Bildhauer et al., 2022; Scala, 2022). They largely fail to account for underlying factors that affect how menstruators experience their menstrual cycles, such as stigma and taboos, and pain, distress and anxiety. We demonstrated that the Brazilian policy reproduced the same results. Yet, we observed that in Brazil the innovative policy language and the significant role of activists in formulating more comprehensive demands points towards a promising scenario for the future. Activists in Brazil articulated an agenda for menstrual health and dignity that is far more comprehensive than seen in many other countries—even if the framing is not yet fully cohesive and compelling.

To advance broader conceptions, we argue that policies in Brazil could benefit from a more open dialogue with civil society. The menstrual movement has been gaining momentum as menstruation becomes more common in public debates and the media. Activists have acquired knowledge of the experiences and needs of menstruators by improving menstrual literacy which could inform storytelling as part of the framing process. Given that menstruation is a near-universal experience for half of the population, such stories have the potential to resonate with other people’s experiences. Activists have demonstrated their capacity to envision policies aligned with the broader understandings of menstrual health and dignity. They can influence the public debate with framings that promote a more positive view of menstruation. Framings such as menstrual dignity that encompass “more than pads” and connect with claims for rights and justice, can contribute to strengthening policies for menstrual literacy, for example. For these framings to find resonance, they need to be made compelling and persuasive.

Further research can explore the influence of framing on policymaking in other contexts. In Latin America, where framings of menstrual dignity and justice are prevalent among social movements, future research can explore if these found resonance in processes of policymaking. At a global level, these discussions can inform the analysis of the framing and shaping of demands, advocacy, and mobilization efforts to change the lives of menstruators. Understanding if and how compelling framings are (re)produced contributes to opening paths for more comprehensive solutions to address menstrual needs.

The political and social debates around menstruation in Brazil can potentially overcome the current limitations of problem representation. The novel terminology introduced by the legislation, that centers menstrual health and dignity, and mentions menstrual education, demonstrates the potential to expand the agenda beyond the material solution of product distribution. This “more than” needs to be framed persuasively to expand menstrual education and provide comprehensive health care. When Bolsonaro’s veto of the original legislation catapulted menstruation onto the national agenda, menstrual policymaking in Brazil already took an unexpected turn. In the context of a government more open to dialogue with social movements, activists are now focused on pushing for another turn—one that advances their agenda and narratives to ensure menstrual health and dignity for all.
